# Convalescent plasma therapy in critically ill coronavirus disease 2019 patients with persistently positive nucleic acid test, case series report

**DOI:** 10.1097/MD.0000000000021596

**Published:** 2020-09-04

**Authors:** Min Wang, Xiaohong Yang, Fang Yang, Xinxin Zhu, Zhibing Sun, Peiling Bao, Yimin Yan

**Affiliations:** a Medical College of Wuhan University of science and technology, Wuhan; b Department of Internal Medicine; c Blood Purification Center, Xiaogan Hospital Affiliated to Wuhan University of Science and Technology, The Central Hospital of Xiaogan, Xiaogan, Hubei, China.

**Keywords:** case series, convalescent plasma therapy, coronavirus disease 2019, convalescent plasma therapy, nucleic acid test

## Abstract

**Introduction:**

Globally, the coronavirus disease 2019 (COVID-19) is still spreading rapidly. At present, there are no specifically approved therapeutic agents or vaccines for its treatment. Previous studies have shown that the convalescent plasma therapy (CPT) is effective in patients with COVID-19. However, its efficacy in patients with persistently positive nucleic acid test is unknown.

**Patient concerns:**

In this report, we present the clinical data of 5 critically ill COVID-19 patients admitted, between January 16 and February 26, 2020, in intensive care unit of Xiaogan Central Hospital.

**Diagnosis and interventions:**

All these patients had a persistently positive nucleic acid test and received CPT. All 5 patients had severe respiratory failure, and thus, required invasive mechanical ventilation. The median time from the onset of symptoms to initiating the CPT was 37 (Interquartile range, 34-44) days.

**Outcomes:**

Only 2 patients were cured and subsequently discharged, while 3 patients succumbed due to multiple organ failure.

**Conclusion:**

The time of initiating the CPT may be an important factor affecting its efficacy, and its therapeutic effect in the treatment of COVID-19, in the late stage, is limited.

## 1 Introduction

The coronavirus disease 2019 (COVID-19) is caused by a new severe acute respiratory syndrome coronavirus 2. Due to its highly transmissible nature, it has rapidly spread around the world.^[1]^ At present, there is neither a preventive measure, in the form of an effective vaccine, nor a curative, specifically approved, antiviral agent.^[2]^ However, convalescent plasma therapy (CPT) has been successfully used to treat the severe acute respiratory syndrome (SARS), Middle East respiratory syndrome (MERS), and H1N1 influenza, and has been found to be an effective measure in reducing the mortality amongst the critically ill patients.^[3]^ In addition, studies have shown that the efficacy of CPT is better in the early stage of disease (within 14 days).^[4]^

However, in the early stage of COVID-19 outbreak, the sources of convalescent plasma (CP) were limited, and thus, the CPT was difficult to carry out. Based on the clinical practice, in this report, 5 critically ill COVID-19 patients with persistently positive nucleic acid test, for more than 30 days, were treated with CP, in the middle and late stages of the disease. Here, we present the retrospective analysis of the clinical data of these 5 critically ill COVID-19 patients and evaluate the effect of CPT.

## 2 Methods

### 2.1 Study population

**Figure 1:**
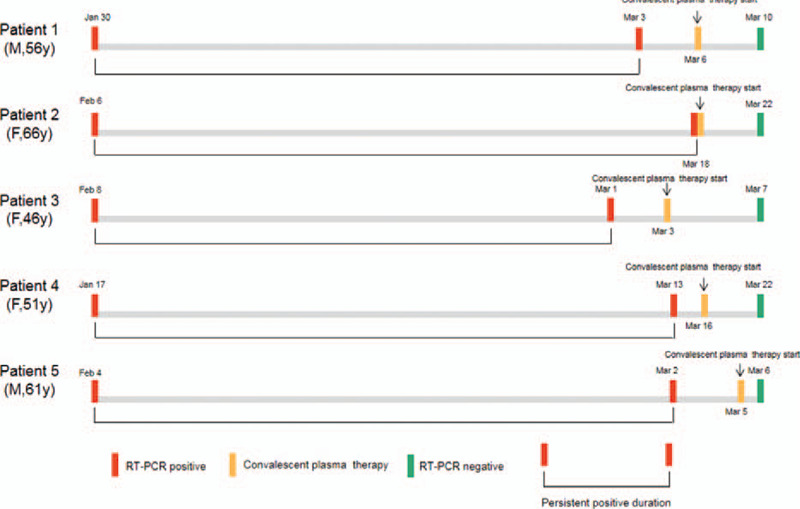
Chronology of detecting positive nucleic acid tests and initiation of CPT in 5 critically ill COVID-19 patients. The red boxes represent the dates of positive nucleic acid tests, the green boxes represent the dates of negative nucleic acid tests, and the yellow boxes represent the dates on which patients received CPT. COVID-19 = coronavirus disease 2019, CPT = convalescent plasma therapy.

Between January 16 and February 26, 2020, a total of 65 critically ill COVID-19 patients were admitted in Xiaogan Central Hospital. Amongst them, 5 (7.69%) patients continued to have a persistently positive nucleic acid test and thus, were included in the study and received CPT (Fig. 1). Both the diagnosis and management of COVID-19, and the collection and treatment standards of CP confirmed to the Diagnosis and Treatment Protocol for Novel Coronavirus Pneumonia (Trial Version 7).^[5]^ This study was conducted according to the principles of Helsinki and approved by the Ethics Committee of The Central Hospital of Xiaogan (No.XGLY2020-03-31). Written informed consent was obtained from all the patients.

### 2.2 CPT

The blood group of all the patients was A+. The CP was derived from the donations of the recently cured patients, for which the antibody titer was above 1:640. According to the principle of cross-matching and blood infusion, only 200 mL of CP was transfused at a time, over a period of 15 min.^[6]^ Amongst 5 patients, 3 received 400 mL and remaining 2 received 1200 mL of CP. The vital signs, laboratory findings, and chest radiographs were collected on the 1st, 3rd, and 7th days after initiating the CPT. The follow-up of all the patients was completed on May 1, 2020.

### 2.3 Statistical analysis

The non-normally distributed data is expressed as median and interquartile range (IQR).

## 3 Results

Amongst 5 patients, 3 were females. The median age was 56 (IQR, 50-62) years. The median time from the onset of symptoms to the diagnosis and initiating the CPT was 6 (IQR, 5-7) days and 37 (IQR, 34-44) days, respectively. The median hospital stay was 51 (IQR, 48–80) days. All the 5 patients had underlying chronic comorbidities, including hypertension and diabetes. Moreover, Patient 1 had both the comorbidities and a chronic history of smoking (Table 1).

**Table 1:**
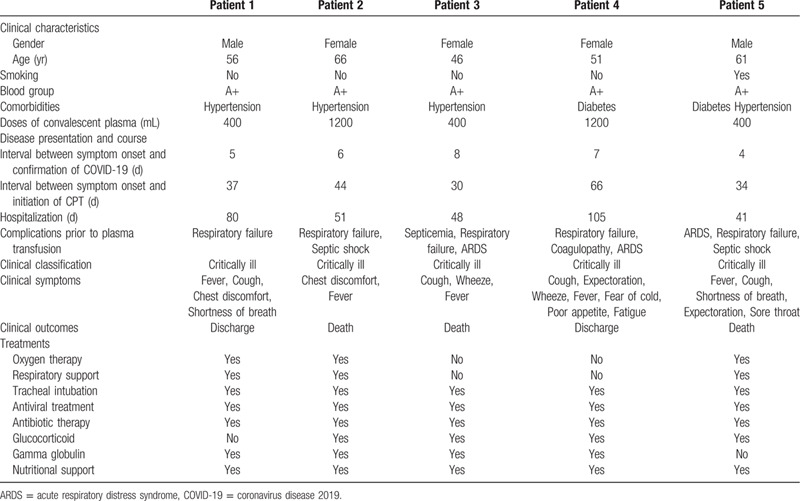
Comparison of clinical characteristics before and after convalescent plasma therapy.

**Figure 2:**
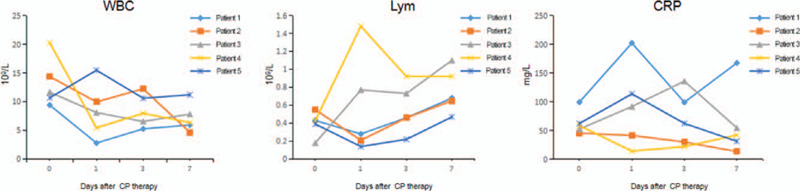
The changes in leukocyte and lymphocyte counts, and CRP levels in 5 critically ill COVID-19 patients on the 1st, 3rd, and 7th day following the initiation of CPT. COVID-19 = coronavirus disease 2019, CPT = convalescent plasma therapy, CRP = C-reactive protein.

**Figure 3:**
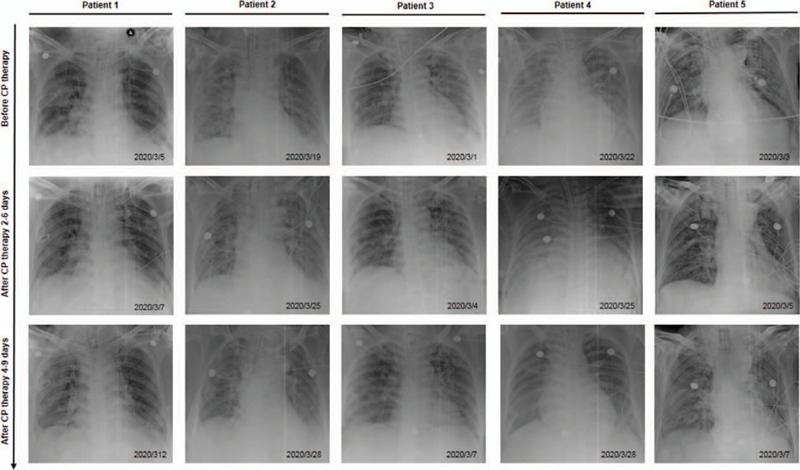
Changes in bedside chest radiographs in 5 critically ill COVID-19 patients before and after receiving the CPT. COVID-19 = coronavirus disease 2019, CPT = convalescent plasma therapy.

Before initiating the CPT, in all the patients, laboratory investigations revealed increased leucocyte and neutrophils counts, and raised levels of C-reactive protein (CRP) and D-dimer. While, the lymphocyte and platelet counts were decreased. In most patients, there were variable degrees of fall and rise in the serum levels of total protein and albumin, and the myocardial enzyme, respectively (Table 2, Fig. 2). The chest radiograph of all the patients revealed patchy, ground-glass opacities (Fig. 3). All of them were admitted to intensive care unit, underwent endotracheal intubation, and required invasive mechanical ventilation. All of them received antibiotics and antiviral agents. Patient 1 and 5 received only gamma-globulin and hormones, respectively. While, the remaining 3 patients received both gamma-globulin and hormones. Additionally, every patients received supportive nutritional treatment (Table 1).

**Table 2:**
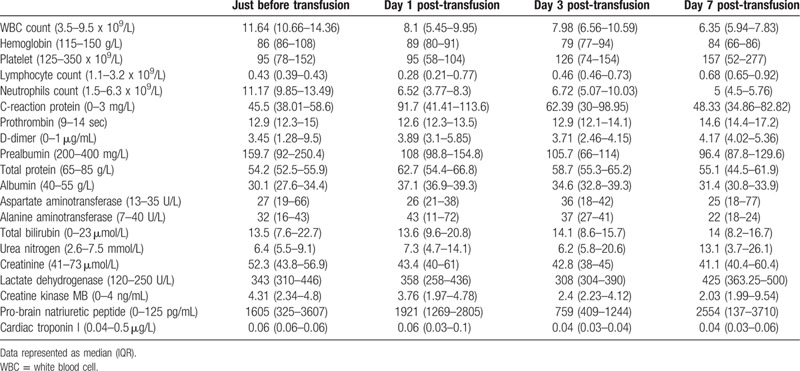
Comparison of laboratory findings before and after convalescent plasma therapy.

Following the initiation of CPT, the changes in vital signs and laboratory findings were observed on Day 1, 3, and 7. Before initiating the CPT, except Patient 2 with fever (38.3°C), other patients had normal body temperature. However, 3 days after initiating the CPT, the temperature of Patient 2 returned to normal (36.2°C), and no significant fluctuation in body temperature was recorded in other patients. In the first 7 days following the initiation of CPT, the leukocyte and neutrophil counts decreased in 3 patients, while the lymphocyte count increased in 4 patients, however, these counts were still above and below the normal range, respectively. Moreover, during this period, only 1 patient demonstrated a downward trend in CRP levels, but it still exceeded the normal range. In addition, liver and kidney functions, and myocardial enzyme did not demonstrate any significant fluctuation (Table 2, Fig. 2). Within 6 days of initiating the CPT, 2 consecutive nucleic acid tests were found to be negative in all the patients. Additionally, 4–9 days following the initiation of CPT, bedside chest radiographs revealed resolution of pulmonary lesion in 3 patients. However, the pulmonary lesions in 1 patient did not change significantly, while in the other patient, it had slightly advanced (Fig. 3). None of the patients developed adverse reactions following the infusion of CPT. Finally, 2 patients were cured and discharged, while the 3 patients succumbed. Analysis of the cause of death in each patient revealed multiple organ failure, disseminated intravascular coagulation combined with gastrointestinal bleeding due to severe infection (Patient 2); acute respiratory distress syndrome, multiple organ dysfunction, and septic shock due to severe infection (Patient 3); and respiratory and circulatory failure due to septic shock (Patient 5).

## 4 Discussion

A study evaluating the efficacy of CPT in influenza A (H1N1, 2009) reported that CPT could result in reduced respiratory viral load and mortality in severely ill patients, and dampen the cytokine response.^[7]^ Another study assessing the role of CPT in patients with SARS revealed a higher discharge rate, on 22nd day of the symptoms onset, amongst the patients receiving CPT.^[8]^ The findings of these studies suggest that the CPT may be beneficial in patients with coronavirus pneumonia. However, the studies involving COVID-19 patients have shown that the CPT is effective, when used during the early stages of infection.^[4]^

In this report, all 5 patients had critical form of COVID-19. Additionally, the nucleic acid test of all these patients remained persistently positive, even after receiving antibiotics, antiviral, and anti-inflammatory agents, along with the symptomatic supportive treatment. After initiating the CPT, most patients maintained normal body temperature and the clinical symptoms improved. Additionally, various investigations revealed decrease in the inflammation index, increase in lymphocyte count, transformation of nucleic acid tests, and decreased pulmonary lesions on chest radiographs. In terms of clinical outcome, 2 patients were cured and discharged, while 3 patients succumbed. Thus, to a certain extent, the use of CPT resulted in an improved patient condition. However, it did not result in reduced mortality rate and the overall outcome was poor.

Studies have demonstrated that in diseases such as SARS, the viremia usually peaks in the first week of infection, while the patients develop an immune response in the second week and this can result in a fatal cytokine storm.^[8,9]^ Although the use of CPT can dampen the cytokine response, there is still a risk of aggravating the immune response, as the CPT is a form of passive immunity involving the administration of pathogen specific antibodies in patients.^[10]^ Thus, in theory, the CPT is more effective in the early stage of the disease. It has been reported that the patients receiving CP, before 14 days following the disease onset, demonstrated a rapid increase in lymphocyte count and a decrease in CRP levels, with remarkable resolution of the pulmonary lesions on computed tomography. However, the patients who received CP after 14 days following the disease onset demonstrated significantly reduced improvement.^[11]^ All the above studies emphasized on the importance of time, while initiating the CPT. In this report, in all the patient, there was a significant delay in initiating the CPT (the median time from the onset of symptoms to the initiation of CPT was 37 days), and this delay may have resulted in poor outcome. Thus, further studies are required to ascertain the ideal time for initiating the CPT.

Additionally, the neutralizing antibody titer (NAT) is reported to affects the efficacy of CPT. Ko et al evaluated the effective of CPT in MERS patients and reported that to achieve an effective treatment outcome the NAT should be more than 1:80.^[12]^ In this report, the antibody titers of plasma used in CPT were above 1:640, but in all the patients the NAT was not detected after the transfusion. Thus, it was not clear whether there were enough neutralizing antibodies in the patients to eliminate the pathogens. Finally, there is absence of uniform standard dose of plasma that needs to be transfused so as to obtain a difference in clinical outcome.

In summary, the CPT is a potential treatment option for the patients with critical form of COVID-19. The CPT with high NAT can significantly reduce the viral load and improve the clinical outcomes. However, the time of initiating the CPT may seriously affect its efficacy. For critically ill COVID-19 patients, initiating the CPT in the early stage may be more efficacious, while its efficacy in the middle and late stages may be much poor.

## Author contribution

**Data curation:** Min Wang, Yimin Yan.

**Investigation:** Fang Yang, Xinxin Zhu.

**Methodology:** Peiling Bao.

**Supervision:** Peiling Bao, Yimin Yan.

**Writing – original draft:** Min Wang, Xiaohong Yang.

**Writing – review & editing:** Yimin Yan.
